# Low muscle mass, low muscle function, and sarcopenia in the urban and rural elderly

**DOI:** 10.1038/s41598-022-18167-y

**Published:** 2022-08-22

**Authors:** Sung Woo Moon, Kwang-Joon Kim, Han Sung Lee, Young Mi Yun, Jong-Eun Kim, You Jin Chun, Chang Oh Kim

**Affiliations:** 1 Division of Geriatrics Medicine, Department of Internal Medicine, Yonsei University College of Medicine, 50 Yonsei-ro, Seodaemun-gu, Seoul, 120-752 Republic of Korea; 2 Division of Integrated Medicine, Department of Internal Medicine, Yonsei University College of Medicine, Seodaemun-gu, Seoul, Republic of Korea; 3 Severance Executive Healthcare Clinic, Yonsei University College of Medicine, Seodaemun-gu, Seoul, Republic of Korea

**Keywords:** Risk factors, Epidemiology, Geriatrics

## Abstract

Health outcomes of the elderly vary between rural and urban areas. Sarcopenia is diagnosed as loss of muscle strength or impaired physical performance, namely “low muscle function” and low muscle mass. Outcomes of low muscle mass and low muscle function are not equal. This study aimed to investigate the prevalence of low muscle mass, low muscle function, and sarcopenia in rural and urban populations and to determine whether regional differences were associated with each of these components. Participants aged ≥ 69 years (n = 2354) were recruited from three urban districts and one rural district in Korea. Low muscle mass was defined by appendicular lean mass using bioelectrical impedance analysis. Low muscle function was defined by handgrip strength and 5-chair stand test. Sarcopenia was defined as low muscle mass plus low muscle function. The prevalence of low muscle function (53.7% vs. 72.8%), and sarcopenia (16.3% vs. 24.4%) were higher in the rural elderly population. Rural residence was associated with low muscle function (OR 1.63; 95% CI 1.13–2.37, P = 0.009), but not with low muscle mass (OR 0.58; 95% CI 0.22–1.54, P = 0.271) or with sarcopenia (OR 1.13; 95% CI 0.63–2.00, P = 0.683). Interventions to detect and improve low muscle function in rural elderly population are needed.

## Introduction

Sarcopenia is a muscle disease rooted in adverse muscle changes that develop across a lifetime^1^. It is currently considered a new geriatric syndrome with multiple risk factors and causes many adverse clinical outcomes^2^. The elderly population is rapidly rising in Korea, and it is estimated to become a super-aged society by 2025^3^. The elderly population in rural areas is growing fast, and approximately half (44.7%) of the rural population is > 65 years^4^. Differences in socioeconomic factors, the physical environment, and health care resources between rural and urban areas weaken the physical, mental, and social health of the elderly, resulting in poor health outcomes^5^. As the health outcomes of older adults vary from rural to urban areas, it is necessary to consider regional differences while studying them^5^.

Only a few studies have explicitly reported the prevalence of sarcopenia in urban and rural populations, and the results have not been consistent. A survey from China^6^ showed that elderly from rural areas were more vulnerable to sarcopenia than that of those from urban areas. In a study reported from Brazil^7^, elderly women from urban areas were more vulnerable to sarcopenia than that of those from rural areas.

Initially, sarcopenia was defined as low muscle mass only^8^, however, now there is a consensus that low muscle mass and low muscle function must be taken together to completely define sarcopenia^1,9^. Further, Asian Working Group for Sarcopenia (AWGS) 2019 defines sarcopenia as low appendicular skeletal muscle mass plus low muscle strength or low physical performance^10^. Although it is known that there is a relationship between muscle mass and muscle function^11^, there are also reports that muscle mass is not synchronous with muscle function. The consequences of muscle mass and muscle function are, therefore, not equal. One study reported that the relationship between muscle mass and muscle function, including handgrip strength is complex and unlikely to be linear^12^. Another study reported that muscle mass was not associated with physical performance in weak older adults^13^. Other investigators have also shown that low muscle strength, not low muscle mass, is associated with disability and mortality^8,14,15^. Therefore, when interpreting the difference between rural and urban areas, researchers should compare not only the diagnosis of sarcopenia but also low muscle mass and low muscle function.

The Korean urban–rural elderly (KURE) study includes the measurements of bioelectrical impedance analysis (BIA), handgrip strength (HGS), and 5-times chair standing test (5-CST) and therefore can be used not just to identify sarcopenia but also to evaluate muscle mass, muscle strength, and physical ability individually. This study aimed to report the prevalence of low muscle mass, low muscle function, and sarcopenia from the KURE study data and evaluate whether regional differences were associated with low muscle mass, low muscle function, and sarcopenia.

## Methods

### Study design and population

In 2012, the Korean government (Centers for Disease Control and Prevention and the Ministry of Health and Welfare, Korea) collected a large prospective cohort of community-dwelling older adults aged ≥ 65 years for a study named KURE. The purpose was to conduct a cohort study to identify the epidemiologic characteristics, physical performance, laboratory and imaging biomarkers, and incidence of age-related diseases concerning both clinical and social aspects in an elderly population^16^.

**Figure 1:**
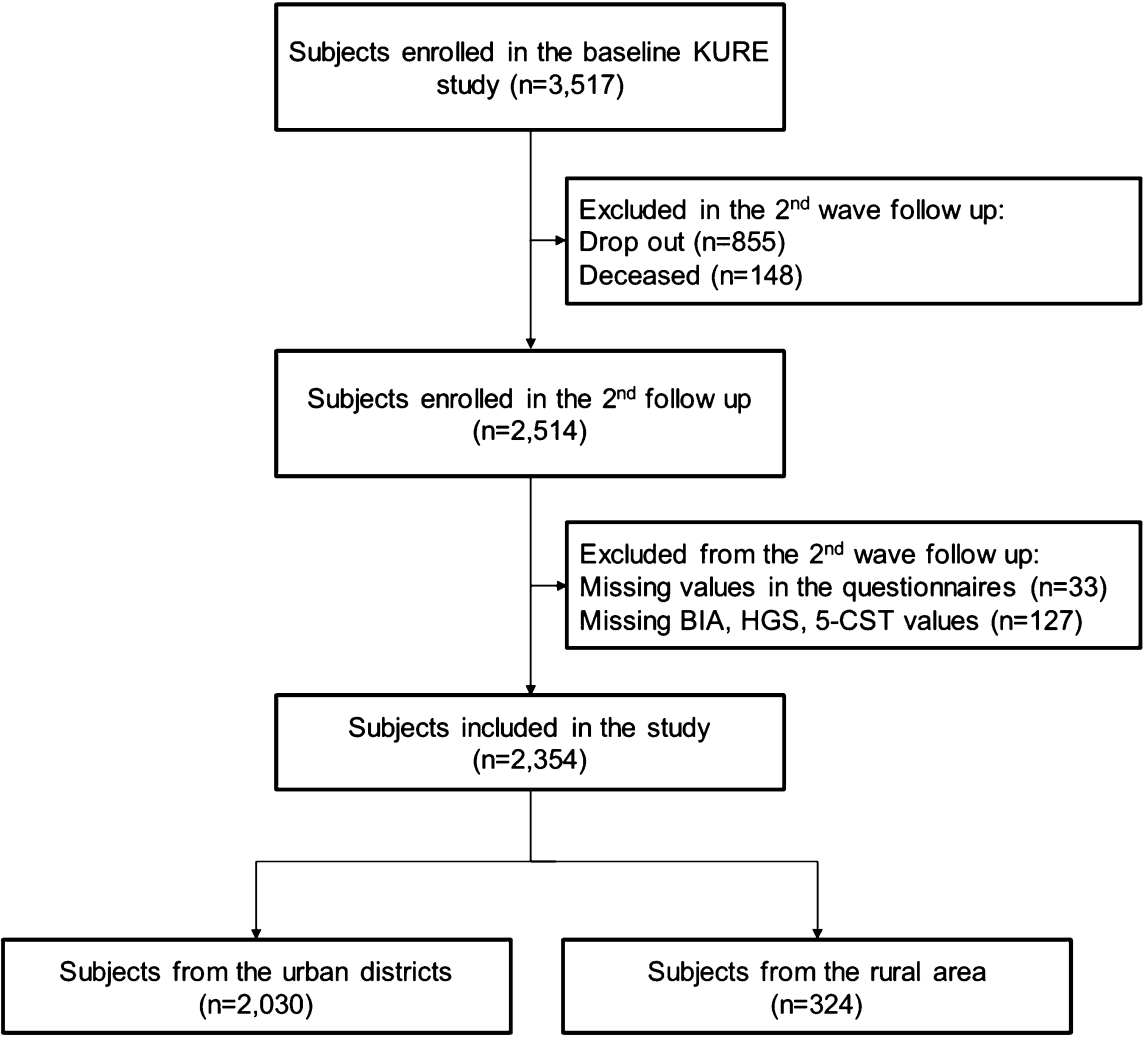
Flow diagram of the participants who were included in the study. *BIA* bioelectrical impedance analysis, *HGS* handgrip strength, *5-CST* 5-times chair standing test (5-CST).

In the KURE study, participants aged ≥ 65 years were initially recruited from three urban districts of Seoul (Mapo-gu, Seodaemun-gu, and EunPyeong-gu) and one rural area of Incheon (Ganghwa) via hospital visits, contact with local government health facilities, and promotion posters. The KURE study group performed a baseline survey between 2012 and 2015, the second round for a 4-year follow-up was carried out between 2016 and 2019, and the third round for an 8-year follow-up is being conducted between 2020 and 2023. This study used the second round of follow-up data because measurement of BIA was not mandatory in the baseline study, and therefore only 52% underwent BIA. Of the 3517 participants enrolled for the baseline study, 2514 people participated in the second round of follow-up (drop out, n = 855; deceased, n = 148) after four years. Among the 2514 participants, 160 were further excluded from analysis for the following reasons: missing values in the questionnaires (n = 33); refused to participate in the BIA, HGS, 5-CST assessment, refuse to participate or data not available (n = 127). Finally, a total of 2,354 participants aged ≥ 69 years were included in the analysis (Fig. 1).

Informed consent was obtained from all participants before the survey and physical examination. Participants took trained interviewer-assisted questionnaires and underwent physical examinations. Data regarding age, sex, body mass index (BMI), educational status, monthly income, smoking status, underlying diseases, and presence or absence of regular physical activity were collected. The academic levels were categorized into elementary school or below, middle school, and high school or above. Physical activity was assessed through International Physical Activity Questionnaires (IPAQ) questionnaires and regular physical activity meant intensive exercise ≥ 75 min/week or aerobic exercise ≥ 150 min/week according to the recommendations by the World Health Organization and American Heart Association^17,18^. Additionally, BIA, HGS, and 5-CST data were collected for all patients. Further details of the KURE study protocol have been described elsewhere^16,19^. This study was performed following the provisions of the Declaration of Helsinki. The institutional review board of Yonsei University Health System, Severance Hospital, approved this cohort study (IRB No.2019-ER6302-02). The appropriate ethics review board approved the study design.

### Collecting BIA, HGS, and 5-CST data

Muscle mass was measured by direct segmental multi-frequency BIA (InBody 720, InBody Co., Ltd., Seoul, Republic of Korea) using a proprietary method of the device. Measurements were performed with the participants in a standing position grasping the handles of the analyzer, thereby ensuring contact with a total of eight electrodes (four sensors for the feet and four for the hands). The system separately measured the impedance of the participants’ right arm, left arm, trunk, right leg, and left leg at six different frequencies (1, 5, 50, 250, 500, and 1000 kHz). Appendicular limb mass (ALM) was calculated as the sum of the lean muscle mass in the bilateral arms and legs. To measure hand grip strength (HGS), participants performed two trials for each hand alternately using a digital hand dynamometer (T.K.K 5401, Takei Scientific Instruments Co., Ltd., Tokyo, Japan) with the elbow flexed at 90°. The participants were instructed to squeeze the grip continuously with full force for at least 3 s and were requested to neither swing the grip dynamometer nor hold their breath during the test^20^. A resting interval of at least 30 s was allowed between each measurement^20^. HGS was defined as the maximally measured grip strength among the four measurements. To measure 5-chair stand test (5-CST), participants were instructed to stand up and sit down five times from a chair as fast as possible; time taken during the test was recorded in seconds^21^.

### Definition of low muscle mass, low muscle function, and sarcopenia

According to the recently recommended diagnostic algorithm of AWGS, sarcopenia was defined as low muscle mass plus low muscle strength or impaired physical performance. Appendicular limb muscle mass (ALM)/height^2^ < 5.7 kg/m^2^ in women and < 7.0 kg/m^2^ in men were regarded as low muscle mass^10^. Low muscle function was defined as low muscle strength or impaired physical performance. Low muscle strength was determined as HGS < 18 kg in females and < 28 kg in males measured by a manual digital dynamometer^10^. Impaired physical performance was described as ≥ 12 s in 5-CST^10^.

### Classification into subgroups

Following the recently recommended diagnostic algorithm of AWGS, participants were classified into one of the following groups: participants with normal muscle mass and function, participants with low muscle mass but without low muscle function, participants with low muscle function but without low muscle mass, and participants with sarcopenia (low muscle mass and low muscle function).

### Statistical analysis

Descriptive statistics are used to describe the variables being studied as proportions or means with standard deviations. Pearson’s Chi-squared tests were conducted to compare categorical variables between two groups, and student’s t-tests were conducted to compare continuous variables between two groups. Standard logistic regression models in which all predictor variables were entered, were used for multivariate logistic regression analyses to estimate the odds ratios (OR) for sarcopenia, low muscle mass, muscle strength, and physical performance while controlling for potential confounding factors. As participants with low muscle mass, low muscle function, and sarcopenia can overlap, multivariate logistic regression analyses were performed to three subsets of participants separately; to estimate the OR for sarcopenia, following participants are included in the analysis: normal muscle mass and function group and sarcopenia group; to estimate the OR for low muscle mass, following participants are included in the analysis: normal muscle mass and function group and low muscle mass without low muscle function; and to estimate the OR for low muscle function, following participants are included in the analysis: normal muscle mass and function group and low muscle function without low muscle mass group. The following covariates were included in the logistic regression model: regional difference, age, sex, BMI, education level, marital status, monthly income, smoking status, regular physical activity, and past medical history. Variables included in all multivariable analysis were tested for multicollinearity. An adjusted *p*-value < 0.05 was considered statistically significant. All statistical analyses were performed with SPSS version 25.0 (SPSS Inc., Chicago, IL, USA) and the statistical program R version 4.1.2 (http://cran.r-project.org).

## Results

### Baseline characteristics

The characteristics of participants according to regional differences are shown in Table 1. Rural participants were older than urban participants, the proportion of women was lower, and there were more current and ex-smokers (All *P* < 0.05). In addition, those living in rural areas had lower BMI in male participants, educational background, a lower proportion of participants with a history of dyslipidemia, cerebrovascular accident, a lower ratio of participants who exercised regularly, and lower monthly income (All *P* < 0.05). A higher proportion of rural participants had low muscle function (P < 0.001) and sarcopenia (P < 0.001) than urban participants. On the contrary, the proportion of participants with low muscle mass (P = 0.095) were similar between the urban and rural participants.

**Table 1:** Baseline characteristics of the participants according to the regional difference.

	Urban, n = 2030 (86.2%)	Rural, n = 324 (13.8%)	P-value
Sex, female	1410 (69.5%)	180 (55.6%)	< 0.001
Age, years	74.9 ± 4.3	76.1 ± 4.5	< 0.001
Body mass index (kg/m^2^), male	24.2 ± 2.7	23.5 ± 3.0	0.013
Body mass index (kg/m^2^), female	24.4 ± 3.2	24.4 ± 3.1	0.373
Marital status, married	1586 (78.1%)	255 (78.7%)	0.902
**Education level**			< 0.001
Elemantary school or below	893 (44.0%)	238 (73.5%)	
Middle school	379 (18.7%)	43 (13.3%)	
High school or above	758 (37.3%)	43 (13.3%)	
Income (100,000 Korean won/month)	17.1 ± 16.9	10.5 ± 14.9	< 0.001
**Smoking status**			0.036
Current	81 (4.0%)	18 (5.6%)	
Ex-smoker	382 (18.8%)	77 (23.8%)	
Never smoker	1566 (77.2%)	229 (70.7%)	
Hypertension	1189 (58.6%)	190 (58.6%)	1.000
Diabetes	480 (23.6%)	73 (22.5%)	0.724
Dyslipidemia	1008 (49.7%)	105 (32.4%)	< 0.001
Arthritis	554 (27.3%)	78 (24.1%)	0.251
Cerebrovascular accident	137 (6.7%)	12 (3.7%)	0.036
Angina or myocardial infarction	447 (22.0%)	81 (25.0%)	0.251
Malignancy	226 (11.1%)	35 (10.8%)	0.924
Regular exercise (≥ 150 min/week)	1234 (60.8%)	89 (27.5%)	< 0.001
Appendicular limb mass/height^2^, kg/m^2^, male	7.48 ± 0.70	7.36 ± 0.70	0.076
Appendicular limb mass/height^2^, kg/m^2^, female	6.13 ± 0.61	6.05 ± 0.64	0.106
Handgrip strength, kg, male	33.3 ± 6.2	31.1 ± 7.8	< 0.001
Hand grip strength, kg, female	20.5 ± 4.5	18.9 ± 5.0	< 0.001
5-chair stand test, seconds	12.2 ± 4.2	14.4 ± 5.3	< 0.001
Normal muscle mass and function	783 (38.6%)	75 (23.1%)	< 0.001
Low muscle mass^a^	486 (23.9%)	92 (28.4%)	0.095
Low muscle function^b^	1090 (53.7%)	236 (72.8%)	< 0.001
Sarcopenia	330 (16.3%)	79 (24.4%)	0.001

Values are presented as numbers (% of total) or mean ± standard deviations.

^a^Appendicular lean mass/height^2^ < 5.7 kg/m^2^ in females and appendicular lean mass/height^2^ < 7 kg/m^2^ in males.

^b^Handgrip strength < 18 kg in females and < 28 kg in males or 5-time chair stand test > 12 s.

Characteristics of the participants according to the of the subgroups following the AWGS algorithm are shown in Table 2. Participants in low muscle mass without low muscle function group had lowest BMI, highest portion of participants with education level elementary school or below, current smoker and who had regular exercise. Participants in low muscle function without low muscle mass group had highest portion of participants with never smoker, history of hypertension, diabetes, arthritis, and malignancy. Participants in sarcopenia group were oldest, had lowest mean income, and had lowest proportion of participants with urban residence and who exercised regularly. Additionally, the characteristics of participants according to the presence of low muscle mass, low muscle function, and sarcopenia are shown in Supplementary Tables S1, S2, and S3.

**Table 2:** Characteristics of the participants according to the subgroups following the AWGS algorithm.

	Normal muscle mass and function group, n = 858 (36.4%)	With low muscle mass^a^, without low muscle function^b^ group, n = 170 (7.2%)	With low muscle function^b^, without low muscle mass^a^ group, n = 917 (39.0%)	Sarcopenia group, n = 409 (17.4%)
Urban residence	783 (91.3%)	157 (92.4%)	760 (82.9%)	330 (80.7%)
Sex, female	536 (62.5%)	114 (67.1%)	663 (72.3%)	277 (67.7%)
Age, years	73.8 ± 3.8	75.0 ± 4.0	75.3 ± 4.2	77.3 ± 4.5
Body mass index (kg/m^2^), male	24.9 ± 2.3	21.1 ± 2.2	24.9 ± 2.4	21.5 ± 2.4
Body mass index (kg/m^2^), female	25.1 ± 2.8	21.6 ± 2.0	25.6 ± 3.0	22.3 ± 2.7
Marital status, married	726 (84.6%)	131 (77.1%)	680 (74.2%)	304 (74.3%)
**Education level**				
Elementary school or below	325 (37.9%)	56 (32.9%)	511 (55.7%)	239 (58.4%)
Middle school	165 (19.2%)	36 (21.2%)	156 (17.0%)	65 (15.9%)
High school or above	368 (42.9%)	78 (45.9%)	250 (27.3%)	105 (25.7%)
Income (100,000 Korean won/month)	19.4 ± 18.0	19.6 ± 22.3	14.6 ± 15.3	12.6 ± 12.8
**Smoking status**				
Current	30 (3.5%)	11 (6.5%)	33 (3.6%)	25 (6.1%)
Ex-smoker	193 (22.5%)	32 (18.8%)	152 (16.6%)	82 (20.1%)
Never smoker	635 (74.0%)	127 (74.7%)	732 (79.8%)	301 (73.8%)
Hypertension	522 (60.8%)	74 (43.5%)	565 (61.6%)	218 (53.3%)
Diabetes	166 (19.3%)	29 (17.1%)	251 (27.4%)	107 (26.2%)
Dyslipidemia	428 (49.9%)	81 (47.6%)	434 (47.3%)	170 (41.6%)
Arthritis	183 (21.3%)	40 (23.5%)	300 (32.7%)	109 (26.7%)
Cerebrovascular accident	41 (4.8%)	9 (5.3%)	63 (6.9%)	36 (8.8%)
Angina or myocardial infarction	187 (21.8%)	28 (16.5%)	216 (23.6%)	97 (23.7%)
Malignancy	98 (11.4%)	16 (9.4%)	102 (11.1%)	45 (11.0%)
Regular exercise (≥ 150 min/week)	561 (65.4%)	118 (69.4%)	464 (50.6%)	180 (44.0%)
Appendicular limb mass/height^2^ (kg/m^2^), male	7.82 ± 0.51	6.59 ± 0.42	7.66 ± 0.48	6.55 ± 0.36
Appendicular limb mass/height^2^ (kg/m^2^), female	6.40 ± 0.44	5.44 ± 0.23	6.35 ± 0.46	5.32 ± 0.38
Hand grip strength (kg), male	36.2 ± 4.9	32.9 ± 3.1	31.3 ± 6.7	27.9 ± 6.9
Hand grip strength (kg), female	23.1 ± 3.1	21.7 ± 2.5	19.4 ± 4.6	16.4 ± 4.0
5-chair stand test, seconds	9.5 ± 1.5	9.4 ± 1.7	14.7 ± 4.4	12.5 ± 4.5

Values are presented as numbers (% of total) or mean ± standard deviations.

^a^Appendicular lean mass/height^2^ < 5.7 kg/m^2^ in female and appendicular lean mass/height^2^ < 7 kg/m^2^ in male.

^b^Handgrip strength < 18 kg in female and < 28 kg in male and/or 5-time chair standing test > 12 s.

### Variables related to low muscle mass, low muscle function, and sarcopenia

Table 3 show the relationship between the covariates and low muscle mass, low muscle function, and sarcopenia in the respective multivariate logistic regression analyses. Age, BMI, education status of high school or above, and regular exercise were independently associated with sarcopenia. Rural residence (Odds Ratio (OR) 0.58; 95% confidence interval (95% CI) 0.22–1.54, *P* = 0.271) was not associated with low muscle mass. Age and BMI were independently associated with low muscle mass. Rural residence (OR 1.63; 95% CI 1.13–2.37, *P* = 0.009) was related with low muscle function. Age (OR 1.10; 95% CI 1.07–1.13, P < 0.001), education status of high school or above (OR 0.63; 95% CI 0.49–0.81, P < 0.001), history of diabetes (OR 1.45; 95% CI 1.12–1.86, P = 0.004) and arthritis (OR 1.54; 95% CI 1.20–1.96, P = 0.001), and regular exercise (OR 0.74; 95% CI 0.59–0.92, P = 0.007) were independently associated with low muscle function. Rural residence (OR 1.13; 95% CI 0.63–2.00, *P* = 0.683) was not associated with sarcopenia. Age, body mass index, education status of high school or above, income, history of diabetes, and regular exercise were independently associated with sarcopenia. We did not find any correlation between marital status, smoking status hypertension, dyslipidemia, angina, myocardial infarction, or malignancy and muscle mass, muscle function and low muscle mass, low muscle function, and sarcopenia.

**Table 3:** Relationship between covariates and low muscle mass, low muscle function, and sarcopenia from the respective multivariate logistic regression analyses.

Variables	Low muscle mass^a^		Low muscle function^b^		Sarcopenia^c^	
	OR (95% CI)	*P*-value	OR (95% CI)	*P*-value	OR (95% CI)	*P*-value
Rural residence	0.58 (0.22–1.54)	0.271	1.63 (1.13–2.37)	0.009	1.13 (0.63–2.00)	0.683
Age, years	1.12 (1.06–1.19)	< 0.001	1.10 (1.07–1.13)	< 0.001	1.22 (1.17–1.27)	< 0.001
Sex, female	1.19 (0.58–2.45)	0.641	1.32 (0.93–1.89)	0.122	1.48 (0.82–2.68)	0.194
Body mass index (kg/m^2^)	0.49 (0.44–0.55)	< 0.001	1.02 (0.98–1.06)	0.270	0.57 (0.52–0.62)	< 0.001
**Education level**						
Elementary school or below	Reference	–	Reference	–	Reference	
Middle school	1.14 (0.63–2.08)	0.659	0.77 (0.58–1.03)	0.074	0.69 (0.44–1.10)	0.115
High school or above	0.75 (0.44–1.28)	0.294	0.63 (0.49–0.81)	< 0.001	0.48 (0.32–0.73)	0.001
Income (100,000 Korean won/month)	1.02 (0.90–1.15)	0.793	0.95 (0.88–1.01)	0.116	0.87 (0.76–0.99)	0.038
Diabetes	0.93 (0.53–1.64)	0.812	1.45 (1.12–1.86)	0.004	1.55 (1.05–2.28)	0.028
Arthritis	1.24 (0.73–2.11)	0.426	1.54 (1.20–1.96)	0.001	1.44 (0.96–2.14)	0.076
Regular exercise	1.19 (0.73–1.92)	0.488	0.74 (0.59–0.92)	0.007	0.43 (0.30–0.61)	< 0.001

Following covariates were included in the logistic regression model: regional difference, age, sex, body mass index, education level, marital status, monthly income, smoking status, regular physical activity, and past medical history (hypertension, diabetes, dyslipidemia, arthritis, cerebrovascular accident, angina or myocardial infarction, and malignancy).

*OR* odds ratio, *95% CI* 95% confidence interval.

^a^Following participants are included in the analysis: normal muscle mass and function group and low muscle mass group. Appendicular lean mass/height^2^ < 5.7 kg/m^2^ in female and appendicular lean mass/height^2^ < 7 kg/m^2^ in male.

^b^Following participants are included in the analysis: normal muscle mass and function group and low muscle function group. Handgrip strength < 18 kg in female and < 28 kg in male and/or 5-time chair standing test > 12 s.

^c^Following participants are included in the analysis: normal muscle mass and function group and sarcopenia group.

## Discussion

This study investigated the prevalence of low muscle mass, low muscle function, and sarcopenia in rural and urban participants and their relationship with regional differences. We report the prevalence of low muscle mass, low muscle function, and sarcopenia in the study population, and we identify a range of factors associated with these three parameters. Among the factors, rural residence was associated with low muscle function. Whereas rural residence was neither associated with low muscle mass nor sarcopenia.

Rapid aging is observed in many parts of the world^22^. This trend is associated with growing global aging rates in rural populations, which are anticipated to increase the demand for health and social care services in non-metropolitan regions^23^.

In this study, the prevalence of sarcopenia was 24.4% in rural elderly and 16.3% in urban elderly. Only a few studies have explicitly compared sarcopenia in urban and rural residents. Gao et al. reported that the prevalence of sarcopenia was 13.1% among the rural population and 7.0% among the urban population by measuring muscle mass via calf circumference^6^. Xin et al. reported that the prevalence of sarcopenia was 21.7% among the rural population and 9.4% among the urban population^24^. Letícia et al. said that the prevalence of sarcopenia was 0.7% among the rural population and 5.7% among the urban population, but the study only included female participants^7^. The differences in the prevalence may be related to age, ethnicities, social and cultural backgrounds, and different measurement methods used.

In 2019, AWGS introduced “possible sarcopenia”, which is characterized by low muscle strength or reduced physical performance^10^. And In 2018, the European working group on sarcopenia in older people introduced “probable sarcopenia”, which is defined by detecting low muscle strength^1^. The diagnosis of probable sarcopenia and possible sarcopenia commonly does not necessarily require low muscle mass. More attention should be paid to loss of muscle strength and reduced physical performance in the rural elderly. European working group on sarcopenia in older people uses low muscle strength as the primary parameter of sarcopenia and muscle strength is presently the most reliable measure of muscle function^1^. They are better than muscle mass in predicting adverse outcomes, markers of frailty^25^, and are an essential indicator of future activities of daily living^26^, and can cause a negative outcome^27,28^.

In this study, rural residence was a risk factor for low muscle function. One reason may be that the physical activity status of rural population is not superior to urban population. Although higher proportion of rural population are more engaged in long hours of heavy physical work such as agricultural work, than the urban population, one study reported that urban elderly have higher self-care and physical activity rates, including flexibility exercises, and muscle strength exercises than the rural elderly^29^. Other reason may be the dietary difference between the urban and rural populations. Adequate nutrition is an important factor to prevent frailty in older adults and a lack of protein intake can lead to frailty, including muscle loss, bone weakness, and reduced immunity^5^. In a study, rural residents’ diet included low intakes of fruits, eggs, fish and seafood^5^. Similarly, another study reported that energy, protein and lipid from animal sources, minerals, and vitamin intake were lower in rural participants^30^.

In this study, BIA, HGS, and 5-CST results were collected to assess the relative muscle power. Alcazar et al. had introduced a simple formula (absolute 5 repetition chair sit to stand power (W) = body weight (kg) × 0.9 × 9.81 × [height (m) × 0.5 − chair height (m)] divided by 5-CST time (s) × 0.1) as a more clinically reactive tool to assess functional trajectory in older people than the classical 5-CST^31^. Another report described that 5 repetition chair sit to stand power test results adjusted by weight^2^ were related to functionality and performance measures more than to sarcopenia^32^. Calculating absolute 5 repetition chair sit to stand power from our data, the values were also higher in urban population than rural population (176.1 ± 1.5 W vs 147.3 ± 3.8 W, P < 0.001).

Exercise and nutritional and drug interventions are commonly being tried for sarcopenia prevention and treatment. But their effect on muscle mass, strength, and physical performance are not equal. In a systematic review, exercise interventions appeared to have a role in increasing muscle strength and improving physical performance. However, they do not seem to increase muscle mass consistently^33^. In another systematic review, although the evidence level was low, nutrition intervention improved knee extension strength. Still, there was no significant effect of nutritional intervention on muscle mass, grip strength, walking speed, and timed up and go test results^34^. The latest evidence shows that vitamin D supplementation and intake of n-3 polyunsaturated fatty acids may benefit body function, muscle quality, and strength^35,36^. Selective androgen receptor modulators increased muscle mass but did not translate to power or physical performance improvement^37^. Through the results of our study, different intervention strategies may be helpful in rural and urban areas to prevent and treat sarcopenia.

The major strength of this well-performed cohort study is that the urban and rural populations were compared not only by the prevalence of sarcopenia but also its components through accurate diagnostic standards, which were redefined recently^10^. The study revealed that screening and intervention are needed for the rural population.

Our study has several limitations. First, due to the cross-sectional study design, any inference on causality could not be made. However, as the third wave for the 8-year follow-up is being conducted in the KURE study, we expect to report additional results. Second, since the recruitment of participants for the KURE study was based on voluntary application, the selection of participants was not random and the possibility of healthy user bias cannot be ruled out, which might lower the prevalence of sarcopenia and partially explain the relatively low prevalence of smokers. However, the validity of reported prognostic factors, such as low BMI, education, and income, were confirmed in this study population, which supports the present findings. Third, due to the study design, recommended methods to measure muscle mass (dual-energy X-ray absorptiometry) and physical performance (6-m walk, short physical performance battery) for the diagnosis of sarcopenia by the AGWS were not included in the study. Dual-energy X-ray absorptiometry is not suitable for large-scale surveys on sarcopenia due to the cost, accessibility, and the problem of radiation exposure. However, BIA has shown efficiency for assessing ALM^38^. Further, 5-CST is a reliable and valid clinical tool applied successfully in several studies^39^.

In conclusion, comparing rural and urban elderly, low muscle function is related to rural residence. On the other hand, sarcopenia and low muscle mass were not significantly associated with rural residence. Our study confirmed no difference between the rural and urban populations regarding the diagnostic criteria for sarcopenia. However, additional screening tests and interventions in the rural population are needed for the elderly in rural districts are more vulnerable to low muscle function.

## Supplementary Information

Supplementary Information.

## Data Availability

The National Biobank of Korea manages all data sets and bioresources of the KURE cohort, Korea National Institute of Health, which holds the right to use or share these resources. Researchers interested in the KURE cohort are welcome to contact us (email address: jhkwh@nih.go.kr) for future collaboration.
