# Supplementary material for: Low muscle mass, low muscle function, and sarcopenia in the urban and rural elderly
Source: Sci Rep. 2022 Aug 22;12:14314. doi: 10.1038/s41598-022-18167-y (PMC9395512; doi:10.1038/s41598-022-18167-y)
Supplement: Supplementary file 1 — Supplementary Information. [file 41598_2022_18167_MOESM1_ESM.docx]

**SUPPLEMENTARY INFORMATION**

**Low muscle mass, low muscle function, and sarcopenia in the urban and rural elderly**

**Authors:**

Sung Woo Moon^1,2^, Kwang-Joon Kim^2^, Han Sung Lee^1,2^, Young Mi Yun^1^, Jong-Eun Kim^1^, You Jin Chun^1,3^, Chang Oh Kim^1*^

**Affiliations:**

^1^Division of Geriatrics Medicine, Department of Internal Medicine, Yonsei University College of Medicine, Seodaemun-gu, Seoul, Republic of Korea

^2^Division of Integrated Medicine, Department of Internal Medicine, Yonsei University College of Medicine, Seodaemun-gu, Seoul, Republic of Korea

^3^Severance Executive Healthcare Clinic, Yonsei University College of Medicine, Seodaemun-gu, Seoul, Republic of Korea

**Supplementary Tables**

Table S1. Characteristics of the subjects according to the presence of low muscle mass†

|  | Normal muscle mass,  n=1775 (75.4%) | Low muscle mass†, n=578 (24.6%) | p-value |
| --- | --- | --- | --- |
| Urban residence | 232 (13.1%) | 92 (15.9%) | 0.084 |
| Sex, Female | 1313 (67.0%) | 276 (70.1%) | 0.967 |
| Age, years | 74.6 ± 4.1 | 76.6 ± 4.5 | < 0.001 |
| Body mass index (kg/m^2^), Male | 24.9 ± 2.4 | 21.4 ± 2.3 | < 0.001 |
| Body mass index (kg/m^2^), Female | 25.4 ± 2.9 | 22.1 ± 2.4 | < 0.001 |
| Marital status, married | 1406 (79.2%) | 434 (75.1%) | 0.028 |
| Education level |  |  | 0.270 |
| Elementary school or below | 836 (47.1%) | 294 (50.9%) |  |
| Middle school | 321 (18.1%) | 101 (17.5%) |  |
| High school or above | 618 (34.8%) | 183 (31.7%) |  |
| Income (100,000 Korean won/month) | 17.0 ± 16.8 | 14.7 ± 16.5 | 0.007 |
| Smoking status |  |  | 0.018 |
| Current | 63 (3.5%) | 36 (6.2%) |  |
| Ex-smoker | 345 (19.4%) | 114 (19.8%) |  |
| Never smoker | 1367 (77.0%) | 427 (74.0%) |  |
| Hypertension | 1087 (61.2%) | 291 (50.3%) | < 0.001 |
| Diabetes | 417 (23.5%) | 136 (23.5%) | 0.980 |
| Dyslipidemia | 862 (48.6%) | 251 (43.4%) | 0.035 |
| Arthritis | 483 (27.2%) | 149 (25.8%) | 0.517 |
| Cerebrovascular accident | 104 (5.9%) | 45 (7.8%) | 0.115 |
| Angina or Myocardial infarction | 403 (22.7%) | 124 (21.5%) | 0.528 |
| Malignancy | 200 (11.3%) | 61 (10.6%) | 0.703 |
| Regular Exercise (≥ 150 min/week) | 1025 (57.7%) | 297 (51.4%) | 0.008 |
| Appendicular limb mass/height^2^ (kg/m^2^), Male | 7.75 ± 0.51 | 6.56 ± 0.38 | < 0.001 |
| Appendicular limb mass/height^2^ (kg/m^2^), Female | 6.37 ± 0.46 | 5.35 ± 0.31 | < 0.001 |
| Hand grip strength (kg), Male | 34.1 ± 6.2 | 29.4 ± 6.4 | < 0.001 |
| Hand grip strength (kg), Female | 21.1 ± 4.4 | 18.0 ± 4.3 | 0.002 |
| 5-chair stand test, seconds | 12.2 ± 4.2 | 13.4 ± 4.9 | < 0.001 |
| Low muscle function‡ | 918 (51.7%) | 408 (70.6%) | < 0.001 |

Values are presented as numbers (% of total) or mean ± standard deviations

†Appendicular lean mass/height^2^ < 5.7 kg/m^2^ in female and appendicular lean mass/height^2^ < 7 kg/m^2^ in male

‡Handgrip strength < 18kg in female and < 28kg in male and/or 5-time chair stand test > 12 seconds

Table S2. Characteristics of the subjects according to the presence of low muscle function‡

|  | Normal muscle function,  n=1,028 (73.9%) | Low muscle function‡,  n=1,326 (26.1%) | p-value |
| --- | --- | --- | --- |
| Urban residence | 940 (91.4%) | 1090 (82.2%) | < 0.001 |
| Sex, Female | 650 (63.2%) | 940 (70.9%) | < 0.001 |
| Age, years | 74.0 ± 3.9 | 75.9 ± 4.4 | < 0.001 |
| Body mass index (kg/m^2^), Male | 24.4 ± 2.7 | 23.7 ± 2.9 | 0.001 |
| Body mass index (kg/m^2^), Female | 24.5 ± 3.0 | 24.7 ± 3.2 | 0.284 |
| Marital status, married | 857 (83.4%) | 984 (74.2%) | < 0.001 |
| Education level |  |  | < 0.001 |
| Elementary school or below | 381 (37.1%) | 750 (56.6%) |  |
| Middle school | 201 (19.6%) | 221 (16.7%) |  |
| High school or above | 446 (43.4%) | 355 (26.8%) |  |
| Income (100,000 Korean won/month) | 19. 4 ± 18.8 | 14.0 ± 14.6 | < 0.001 |
| Smoking status |  |  | 0.036 |
| Current | 41 (4.0%) | 58 (4.4%) |  |
| Ex-smoker | 225 (21.9%) | 234 (17.7%) |  |
| Never smoker | 762 (74.1%) | 1,033 (78.0%) |  |
| Hypertension | 596 (58.0%) | 783 (59.0%) | 0.613 |
| Diabetes | 195 (19.0%) | 358 (27.0%) | < 0.001 |
| Dyslipidemia | 509 (49.5%) | 604 (45.6%) | 0.061 |
| Arthritis | 223 (21.7%) | 409 (30.8%) | < 0.001 |
| Cerebrovascular accident | 50 (4.9%) | 99 (7.5%) | 0.010 |
| Angina or Myocardial infarction | 215 (20.9%) | 313 (23.6%) | 0.123 |
| Malignancy | 114 (11.1%) | 147 (11.1%) | 1.000 |
| Regular Exercise (≥ 150 min/week) | 679 (66.1%) | 644 (48.6%) | < 0.001 |
| Appendicular limb mass/height^2^ (kg/m^2^), Male | 7.64 ± 0.66 | 7.28 ± 0.55 | < 0.001 |
| Appendicular limb mass/height^2^ (kg/m^2^), Female | 6.23 ± 0.55 | 6.04 ± 0.64 | < 0.001 |
| Hand grip strength (kg), Male | 35.7 ± 4.8 | 30.1 ± 6.9 | < 0.001 |
| Hand grip strength (kg), Female | 22.9 ± 3.0 | 18.6 ± 4.7 | < 0.001 |
| 5-chair stand test, seconds | 9.4 ± 1.6 | 14.8 ± 4.5 | < 0.001 |
| Low muscle mass† | 170 (16.5%) | 408 (30.8%) | < 0.001 |

Values are presented as numbers (% of total) or mean ± standard deviations

†Appendicular lean mass/height^2^ < 5.7 kg/m^2^ in female and appendicular lean mass/height^2^ < 7 kg/m^2^ in male

‡Handgrip strength < 18kg in female and < 28kg in male and/or 5-time chair stand test > 12 seconds

Table S3. Characteristics of the subjects according to the presence of sarcopenia

|  | Without sarcopenia,  n=1945 (82.6%) | With sarcopenia,  n=409 (16.3%) | p-value |
| --- | --- | --- | --- |
| Urban residence | 1,700 (87.4%) | 330 (80.7%) | < 0.001 |
| Sex, Female | 1,313 (67.5%) | 277 (67.7%) | 0.954 |
| Age, years | 74.6 ± 4.1 | 77.3 ± 4.5 | < 0.001 |
| Body mass index (kg/m^2^), Male | 24.6 ± 2.6 | 21.5 ± 2.4 | < 0.001 |
| Body mass index (kg/m^2^), Female | 25.1 ± 3.0 | 22.3 ± 2.7 | < 0.001 |
| Marital status, married | 1,537 (79.0%) | 304 (74.3%) | 0.012 |
| Education level |  |  | < 0.001 |
| Elementary school or below | 892 (45.9%) | 239 (58.4%) |  |
| Middle school | 357 (18.4%) | 65 (15.9%) |  |
| High school or above | 696 (35.8%) | 105 (25.7%) |  |
| Income (100,000 Korean won/month) | 17.2 ± 17.4 | 12.6 ± 12.8 | < 0.001 |
| Smoking status |  |  | 0.090 |
| Current | 74 (3.8%) | 25 (6.1%) |  |
| Ex-smoker | 377 (19.4%) | 82 (20.1%) |  |
| Never smoker | 1,494 (76.8%) | 301 (73.8%) |  |
| Hypertension | 1,161 (59.7%) | 218 (53.3%) | 0.018 |
| Diabetes | 446 (22.9%) | 107 (26.2%) | 0.178 |
| Dyslipidemia | 943 (48.5%) | 170 (41.6%) | 0.012 |
| Arthritis | 523 (26.9%) | 109 (26.7%) | 0.951 |
| Cerebrovascular accident | 113 (5.8%) | 36 (8.8%) | 0.033 |
| Angina or Myocardial infarction | 431 (22.2%) | 97 (23.7%) | 0.514 |
| Malignancy | 216 (11.1%) | 45 (11.0%) | 1.000 |
| Regular Exercise (≥ 150 min/week) | 1143 (58.8%) | 180 (44.0%) | < 0.001 |
| Appendicular limb mass/height^2^ (kg/m^2^), Male | 7.65 ± 0.60 | 6.55 ± 0.36 | < 0.001 |
| Appendicular limb mass/height^2^ (kg/m^2^), Female | 6.29 ± 0.51 | 5.32 ± 0.38 | < 0.001 |
| Hand grip strength (kg), Male | 34.0 ± 6.0 | 27.9 ± 6.9 | < 0.001 |
| Hand grip strength (kg), Female | 21.1 ± 4.3 | 16.5 ± 4.0 | < 0.001 |
| 5-chair stand test, seconds | 11.9 ± 4.2 | 15.1 ± 4.9 | < 0.001 |

Values are presented as numbers (% of total) or mean ± standard deviations
